# Long‐term outcomes of cutaneous ureterostomy with the aim of stent‐free stoma

**DOI:** 10.1002/bco2.499

**Published:** 2025-02-24

**Authors:** Chul Jang Kim, Masayuki Nagasawa, Eiki Hanada, Kayo Takeuchi, Toshiyuki Ihara, Susumu Kageyama

**Affiliations:** ^1^ Department of Urology Kohka Public Hospital Shiga Japan; ^2^ Department of Urology Shiga University of Medical Science Shiga Japan

**Keywords:** bladder cancer, cutaneous ureterostomy, hydronephrosis, stent, urinary diversion

## Abstract

**Objectives:**

We evaluated post‐surgical renal function and risk factors for renal function deterioration (RFD), defined as a > 25% decrease in the estimated serum creatinine‐based glomerular filtration rate (eGFR), after cutaneous ureterostomy (CU) and collected follow‐up data on hydronephrosis after CU construction.

**Patients and methods:**

CU was performed following radical cystectomy in 46 patients (90 renal units [RUs]) with a minimum follow‐up period of 12 months. The median follow‐up period was 102.1 months. The stoma was created using the Toyoda method. A surgical stabilization step for the abdominal tunnel of the ureters was added. Post‐surgical changes in renal function and hydronephrosis were reviewed.

**Results:**

At the end of follow‐up, RFD was observed in 19 (41.3%) of 46 patients. The 5‐ and 10‐year RFD‐free survivals were 61.3% and 47.2%, respectively. Seventy‐six RUs (84.4%) exhibited no hydronephrosis, whereas six RUs (6.7%) in six patients progressed to atrophic kidneys. Stent catheters were inserted in eight RUs (8.9%) in six patients. After excluding 10 patients with progression to atrophic kidneys (six patients) or ureteral obstruction attributable to retroperitoneal lymph node metastasis by cancer progression (four patients), RFD was identified in 13 (36.1%) out of 36 patients. These patients were categorized into Group 1 (without RFD, 23 patients) and Group 2 (with RFD, 13 patients). Stent insertion was identified as a significant predictor of post‐surgical RFD by univariate (*p* = 0.001) and multivariate analyses (*p* = 0.001).

**Conclusions:**

RFD was observed in 41.3% patients during follow‐up. We achieved an 84.4% hydronephrosis‐free rate following CU construction. Stent insertion was identified as a significant risk factor for RFD after CU construction.

## 1 INTRODUCTION

Cutaneous ureterostomy (CU) is the simplest of all permanent urinary diversion methods, but it carries a risk of stomal obstruction.^1^ This complication can lead to renal function deterioration (RFD) and the development of chronic kidney disease (CKD), as observed during a long‐term follow‐up study.^2^, ^3^ CKD is an established risk factor for cardiovascular events and mortality.^4^, ^5^ Achieving CU without hydronephrosis might help reduce late complications.^6^ Various attempts have been employed to minimize stomal obstruction of CU.^6^, ^7^, ^8^, ^9^, ^10^, ^11^ We performed CU using the Toyoda method^8^ and incorporated a surgical stabilization step for the abdominal wall tunnel of the ureters^11^ to improve the catheter‐free rate.

In this study, we evaluated postsurgical renal function and the risk factors for RFD after the construction of CU and presented follow‐up data for hydronephrosis in patients who underwent CU.

## 2 MATERIAL AND METHODS

The medical charts and follow‐up data of 50 patients who underwent open radical cystectomy with CU between October 2005 and December 2017 at our hospital were retrospectively reviewed. Of these, 46 patients (90 renal units[RUs]) with at least 12 months of follow‐up were included in this study. Informed consent was obtained from all patients, and the study design was approved by the Research Ethics Committee of our hospital.

The underlying conditions included bladder cancer in 44 patients and bladder cancer after retroperitoneoscopic‐assisted laparoscopic nephroureterectomy with a cuff for unilateral renal pelvic cancer in two patients. The cohort comprised 39 men and 7 women with a mean age of 70.0 ± 7.1 years (range 54–87 years). The mean body mass index (BMI) was 22.6 ± 3.1 kg/m^2^ (range 16.7–31.1). The pathological stages of bladder cancers were 0is in six patients (13.0%), I in 11 patients (23.9%), II in 18 patients (39.1%), III in 10 patients (21.7%) and IV in one patient (2.2%).

CU was constructed after open radical cystectomy. In 44 patients (95.7%), both ureters were used to create a unilateral stoma (right side in 32 patients, left side in 12 patients). In the remaining two patients, a single ureter was used to construct CU (right side and left side in one patient each), because of a previous retroperitoneoscopic‐assisted laparoscopic nephroureterectomy with a cuff for renal pelvic cancer. The unilateral stoma was successfully created in all 46 patients (right side in 33 patients, left side in 13 patients). The ureteral course was created using the surgical procedure described by Straffon et al.,^12^ whereas the stoma was constructed using the Toyoda method.^8^ To prevent ureteral stenosis in the abdominal wall, a stabilization step for the abdominal wall tunnel of the ureters was added.^11^ After surgery, a 6‐Fr single‐J stent was placed in the renal pelvis through the stoma. The single‐J stent was exchanged every 4 weeks and removed 3 months post‐surgery, because of unstable and obstructive stomal conditions in the early post‐surgical phase.^13^, ^14^ Following stent removal, excretory urography was performed from 2 to 4 days to assess stomal conditions. Patients underwent follow‐up in our hospital every 3 months for the first 2 years and every 6 months thereafter.

Chronic hypertension is defined as the administration of one or more anti‐hypertensive medications. Acute pyelonephritis was defined as hospitalization for either febrile episodes or flank tenderness with a positive urine culture (>1 × 10^5^ colony‐forming units), excluding other causes of fever. The four‐grade system was used to evaluate hydronephrosis.^15^ The estimated serum creatinine‐based glomerular filtration rate (eGFR) was calculated according to the formular recommended by The Japanese Society of Nephrology as follows: eGFR (mL/min/1.73 m^2^) = 194 × serum‐creatinine^‐1.097^ × age^‐0.287^ (×0.739, if female).^16^ RFD was defined as a > 25% decrease in eGFR relative to that prior to surgery as previously described.^17^ Late RFD was also defined as a reduction in the GFR of > 1 ml/min/1.73 m^2^ annually.^18^, ^19^

Statistical analyses were performed using IBM SPSS Statistics version 25 (IBM, Armonk, NY, USA) with significance defined by *p* < 0.05. The RFD‐free survival was estimated using the Kaplan–Meier method, and distributions were compared using the log‐rank test. Differences between the two groups were examined using Student's *t*‐test, and the association between various parameters and post‐surgical RFD was assessed using the Cox proportional hazards model. The χ^2^ test or Fisher's exact test was used to assess differences between groups.

## 3 RESULTS

The mean follow‐up duration was 102.1 ± 54.2 months (range, 12–212). By the end of the study in June 2023, 16 patients were alive without disease, one patient was alive with disease, 11 patients had died from their disease, one patient was alive with a different type of cancer, 10 patients had died of other causes and 7 patients were transferred to other hospitals because of relocation.

**FIGURE 1:**
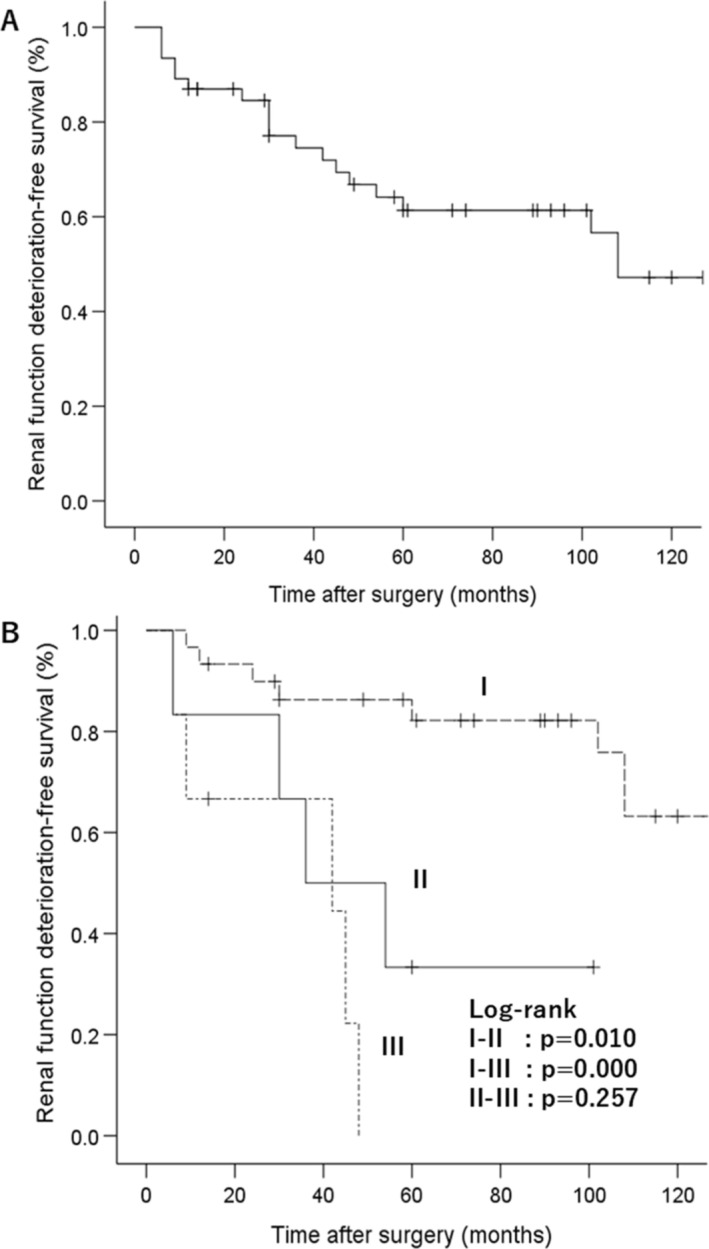
(A) Kaplan–Meier curve presenting the renal function deterioration (RFD)‐free survival in all patients. (B) Kaplan–Meier curve showing the RFD‐free survival among stent‐free patients without hydronephrosis (I, *n* = 30), patients with atrophic kidneys without stent insertion (II, *n* = 6) and stent‐indwelling patients (III, *n* = 6).

During the observation period, the mean eGFR decreased from 67.3 to 56.5 ml/min/1.73m^2^ (15 years after surgery), and RFD was observed in 0 (0.0%), 3 (6.5%) and 19 (41.3%) out of 46 patients at 3 months, 6 months and overall follow‐up, respectively. Three years after surgery, the mean eGFR had significantly decreased compared with the pre‐surgical value (*p* = 0.048). The 5‐ and 10‐year RFD‐free survivals were 61.3% and 47.2%, respectively (Figure 1A).

**FIGURE 2:**
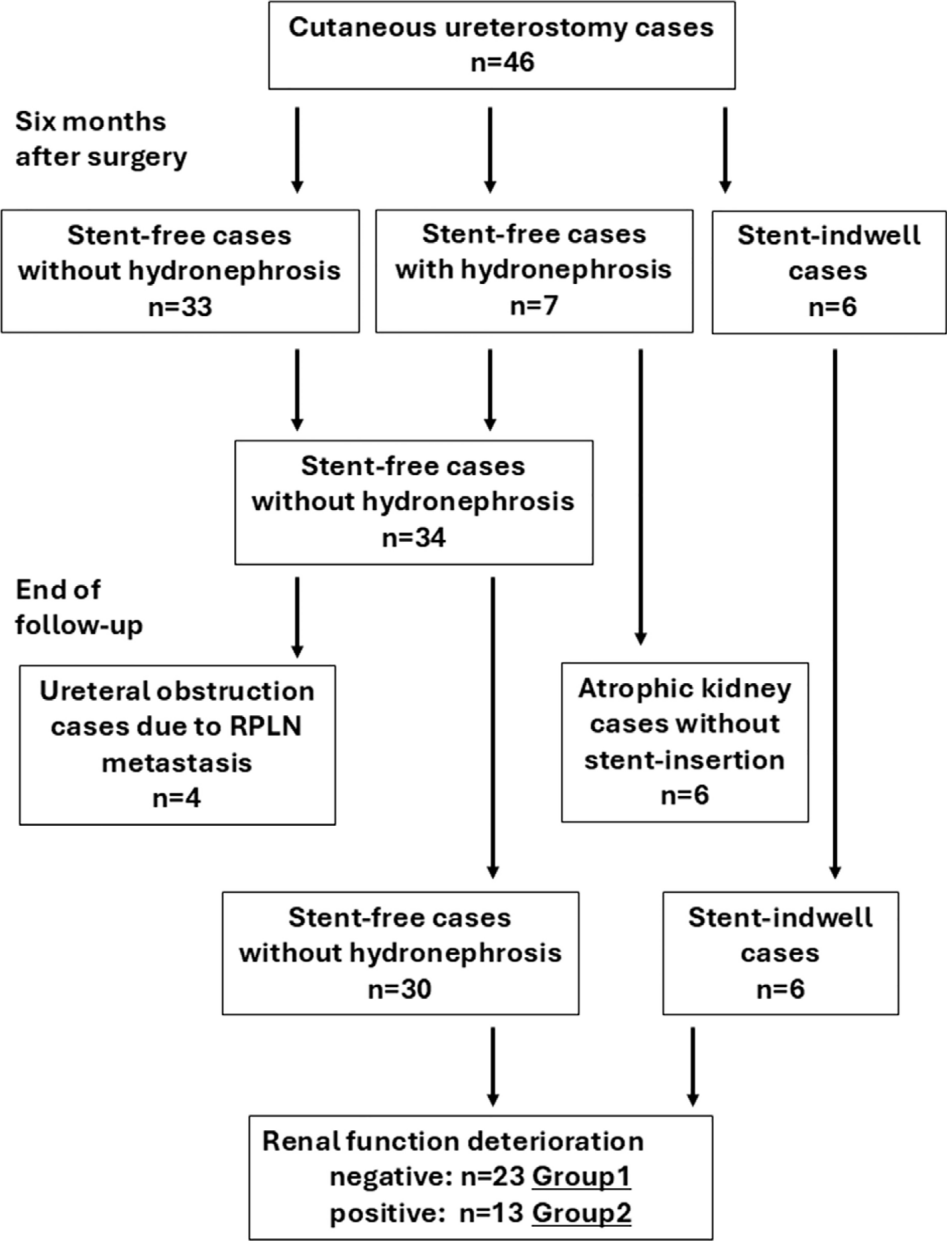
Flow diagram of the upper urinary tract after the construction of cutaneous ureterostomy. RPLN, retroperitoneal lymph node.

Grade 1 or 2 hydronephrosis was present in two RUs (2.2%), being caused by ureteral obstruction attributable to bladder cancer before surgery (Table 1). After the removal of single‐J stents 3 months after surgery, 26 RUs (28.9%) had no hydronephrosis. Hydronephrosis of grades 1, 2 and 3 was present in 18 (20.0%), 32 (35.6%) and 11 RUs (12.2%), respectively. Stent insertions were performed in three RUs (3.3%) in two patients. A flow diagram of the upper urinary tract after CU construction is shown in Figure 2. Six months after surgery, 75 RUs (83.3%) had no hydronephrosis. Hydronephrosis of grades 1 and 2 was present in two (2.2%) and five RUs (5.6%), respectively. Stent insertion was performed in five RUs in four patients. By the end of follow‐up, 76 RUs (84.4%) had no hydronephrosis, whereas six RUs (6.7%) had progressed to atrophic kidneys in six patients. Stent catheters were inserted in eight RUs (8.9%) in six patients. All six RUs in atrophic kidneys were contralateral kidneys in a side relationship between the ureter and stoma. Four (50.0%) out of eight RUs in stent‐inserted patients were contralateral kidneys. Altogether, 10 (71.4%) out of 14 RUs were contralateral kidneys in the progression to atrophic or stent‐inserted kidneys. There was a significant difference between the ipsilateral and contralateral kidneys (*p* = 0.009).

**TABLE 1:** Temporal changes in hydronephrosis following cutaneous ureterostomy.

	Four‐grade system for hydronephrosis *					Atrophic	Stent
	0	I	II	III	IV	Kidney	Insertion
Before surgery	88 (97.8%)	1 (1.1%)	1 (1.1%)	0	0	0	0
Stent removal at 3 months post‐surgery							
3 months post‐surgery	26 (28.9%)	18 (20.0%)	32 (35.6%)	11 (12.2%)	0	0	3 (3.3%)
6 months post‐surgery	75 (83.3%)	2 (2.2%)	5 (5.6%)	0	0	0	8 (8.9%)
At the end of follow‐up	76 (84.4%)	0	0	0	0	6 (6.7%)	8 (8.9%)

The numerical value: renal units (RUs).

* Talner LB: Clinical Urography, 1990.

The significance of several factors as predictors of post‐surgical RFD was examined. According to the progression of the atrophic kidneys, kidneys gradually became atrophic via the persistent hydronephrosis during the follow‐up (Figure 2). It was suggested that stent insertion was essential for preserving renal function in these cases. Additionally, ureteral obstruction attributable to retroperitoneal lymph node (RPLN) metastasis from cancer progression occurred in four patients (eight RUs) after establishing a hydronephrosis‐free condition (Figure 2). A stent‐free stoma was consequently constructed in these patients. After excluding 10 patients with progression to atrophic kidneys (six patients) or ureteral obstruction attributable to RPLN metastasis (four patients), 13 (36.1%) out of 36 patients exhibited RFD. These patients were categorized into Group 1 (23 patients without RFD) or Group 2 (13 patients with RFD, Figure 2). Stent insertion was the only significant predictor of post‐surgical RFD (Table 2). Additionally, stent insertion was also associated with postsurgical RFD in multivariate analysis (Table 3). Conversely, age, sex, presurgical eGFR, acute pyelonephritis, urolithiasis, systemic chemotherapy, hypertension, diabetes mellitus and BMI were not associated with RFD.

**TABLE 2:** Parameters associated with deterioration of renal function in group 1 and group 2.

Variables		Deterioration of renal function		
	Total	Negative (Group 1)	Positive (Group 2)	*p*
*n*	36	23	13	
Age (years)	69.9 ± 6.5	70.0 ± 7.3	69.7 ± 4.7	0.894
Sex				
Male	32	20	12	0.627
Female	4	3	1	
Follow‐up period (months)	111.6 ± 53.6	100.5 ± 57.3	131.1 ± 41.3	0.101
Presurgical eGFR (mL/min/1.73 m^2^)	67.0 ± 16.3	64.5 ± 13.4	71.4 ± 20.2	0.230
Stent insertion				
Positive	6	1	5	0.025
Negative	30	22	8	
Acute pyelonephritis				
Positive	13	9	4	0.617
Negative	23	14	9	
Urolithiasis				
Positive	7	3	4	0.208
Negative	29	20	9	
Systemic chemotherapy				
Positive	12	6	6	0.225
Negative	24	17	7	
Hypertension				
Positive	16	8	8	0.126
Negative	20	15	5	
Diabetes mellitus				
Positive	7	5	2	0.645
Negative	29	18	11	
Body mass index	22.5 ± 3.2	22.2 ± 3.4	22.9 ± 2.9	0.549

Abbreviations: eGFR, estimated serum creatinine‐based glomerular filtration rate.

Values are expressed as the mean ± standard deviation.

**TABLE 3:** Univariate and multivariate analyses of factors associated with the deterioration of renal function in group 1 and group 2.

Variables	Univariate			Multivariate		
	Odds ratio	95% CI	*p*	Odds ratio	95% CI	*p*
Age (≥70 vs. <70 years)	0.703	0.230–2.150	0.536			
Sex (male vs. female)	1.842	0.239–14.194	0.558			
Presurgical eGFR	1.688	0.565–5.043	0.348			
(<60 vs. ≥60 ml/min/1.73 m^2^)						
Stent insertion (+/−)	10.469	0.273–40.130	0.001	10.469	0.273–40.130	0.001
Acute pyelonephritis (+/−)	0.672	0.205–2.195	0.510			
Urolithiasis (+/−)	1.514	0.461–4.974	0.494			
Systemic chemotherapy (+/−)	2.141	0.717–6.395	0.173			
Hypertension (+/−)	0.886	0.297–2.643	0.829			
Diabetes mellitus (+/−)	0.618	0.136–2.801	0.532			
Body mass index	1.506	0.463–4.897	0.496			
(≥25 vs. <25 kg/m^2^)						

Abbreviations: CI, confidence interval, eGFR, estimated serum creatinine‐based glomerular filtration rate.

**FIGURE 3:**
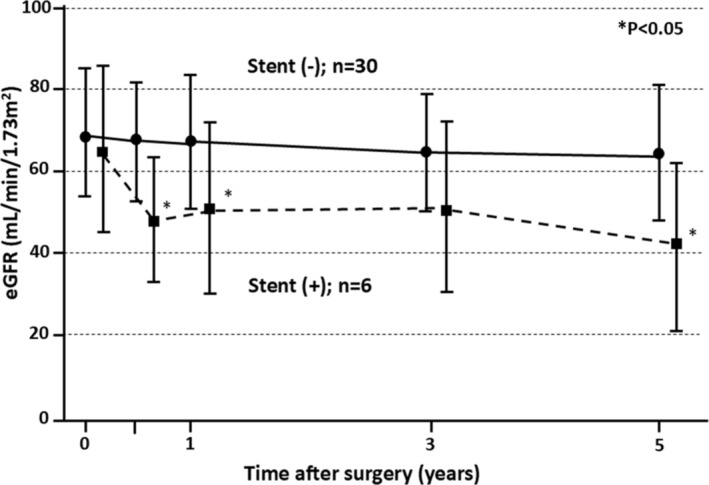
Serial changes in eGFR comparing stent‐free (solid line) and stent‐inserted (dotted line) patients after surgery. Ten patients were excluded because of progression to atrophic kidneys (six patients) or ureteral obstruction caused by retroperitoneal lymph node metastasis attributable to cancer progression (four patients). Asterisks (*) indicate significant differences between stent‐free and stent‐inserted patients (*p* < 0.05). Bars, standard deviation. eGFR, estimated serum creatinine‐based glomerular filtration rate.

We compared the RFD‐free survival among three‐groups which consisted of the stent‐free patients without hydronephrosis (I), the patients with atrophic kidneys without stent insertion (II) and the patients with indwelling stents (III, Figures 1B, and 2). The RFD‐free survival was higher in cohort‐I than in the other two cohorts (*p* < 0.05). The 5‐year RFD‐free survivals in cohorts in I, II and III were 82.2%, 33.3% and 0.00%, respectively (Figure 1B). In addition, the time‐course changes in eGFR were shown in patients with and without stent insertion in Group 1 and Group 2 (Figure 3). Six months after surgery, there was a significant difference in mean eGFR between the stent‐inserted (46.2 ± 16.0 ml/min/1.73 m^2^) and stent‐free patients (67.2 ± 12.9 ml/min/1.73 m^2^, *p* = 0.001). The mean eGFR decreased after surgery in stent‐inserted patients, including a rapid decline of eGFR during the first 6 months after surgery (26% decrease in mean eGFR) and a slow continuous decrease in subsequent years (2.4 ml/min/1.73 m^2^ per year for 4 years). By contrast, similar RFD was not observed in the stent‐free patients. The renal function of the stent‐free patients remained in relatively good condition.

## 4 DISCUSSION

In this study, we demonstrated that the 5‐year and 10‐year RFD‐free survivals after CU construction were 61.3% and 47.2%, respectively. There have been few reports describing long‐term RFD in CU. Two studies found that 46% and 57% of patients who underwent CU procedures revealed RFD during median follow‐up periods of 60 and 99 months, respectively.^3^, ^20^ Our study demonstrated that RFD was observed in 41% of patients during a median follow‐up period of 102 months, indicating that renal function was preserved better in our study than in other studies on CU. A hydronephrosis‐free condition was achieved in 76 (84.4%) of 90 RUs in CU. Previous reports have demonstrated stent‐free rates ranging from 76.9% to 92.4% in CU.^11^, ^14^, ^21^, ^22^, ^23^, ^24^ However, half of these reports did not account for persistent hydronephrosis. Among the studies that did, persistent hydronephrosis without the need for intervention was observed in approximately 10%–20% patients.^11^, ^23^, ^24^ In this respect, our results appear to be superior. Therefore, it is thought that our surgical procedure would contribute to the reduction of stomal obstruction in CU.

Stomal obstruction is a well‐known complication of CU, which is associated with post‐surgical RFD.^3^ Although one report found that stent insertion did not affect RFD in CU,^3^ our study demonstrates that stent insertion is a significant risk factor for RFD in CU during long‐term follow‐up. This result indicated that the renal injury persisted even after the stent insertion for stomal obstruction of CU. Renal function, as measured by eGFR, declined rapidly after surgery in stent‐inserted patients, as illustrated by a biphasic decline in eGFR (Figure 3). Similar patterns of renal function decline have been observed following radical cystectomy with urinary diversion, with a rapid decline during the first year and a slower, continuous decline thereafter.^19^, ^20^, ^25^ However, this biphasic decline was not observed in stent‐free patients, whose renal function remained stable. Ureteroenteric strictures are the most important cause of post‐surgical early renal impairment after radical cystectomy with urinary diversion.^19^ We suggest that stomal obstruction of CU is comparable to ureteroenteric stricture as a significant risk factor for early RFD. If a hydronephrosis‐free condition can be achieved, then CU could be a useful permanent urinary diversion comparable to the ileal conduit.

In the early post‐surgical period, the stoma of CU is edematous and unstable,^13^, ^14^ resulting leading to hydronephrosis, as determined by excretory urography. In this study, hydronephrosis was observed in 64 (71.1%) out of 90 RUs 3 months after surgery. By the end of the follow‐up period, 76 (84.4%) of 90 RUs had no hydronephrosis, indicating that most stomas were not obstructed. Unnecessary stent insertion may sometimes occur in patients with hydronephrosis and acute pyelonephritis. Conversely, six (85.7%) of seven RUs with grade 1 (two RUs) or grade 2 (5 RUs) hydronephrosis at 6 months after surgery became atrophic by the end of follow‐up. Prompt stent insertion in cases of grade 2 hydronephrosis at 6 months after surgery is therefore recommended to reduce further kidney damage.

In our study, eight (17.4%) out of 46 cases experienced urolithiasis, which is associated with an increased risk for CKD in symptomatic kidney stone formers.^26^ Urolithiasis was not identified as a significant risk factor for post‐surgical RFD in univariate and multivariate analyses. Although stent insertion might be expected to increase the risk of urolithiasis and subsequent RFD, all eight cases of urolithiasis occurred in stent‐free patients, and the incidence did not differ significantly between stent‐inserted patients (0/6, 0.0%) and the other patients (8/40, 20.0%, *p* = 0.228). Acute pyelonephritis has also been linked to late renal function decline following radical cystectomy with urinary diversion,^3^, ^19^ but no significant difference in RFD was observed between the patients with or without acute pyelonephritis in this study. Additionally, repeated episodes of pyelonephritis, which have been reported as a risk factor for RFD,^2^ did not influence RFD in this cohort (data not shown).

Our study had some limitations, including its retrospective design, small sample size and variability in patient care. Furthermore, excluding patients without long‐term renal function data from the analysis might have introduced selection bias. Nevertheless, we assumed that it is important to examine the long‐term outcomes of CU at high hydronephrosis‐free rate.

## 5 CONCLUSION

RFD, defined as a > 25% decrease in eGFR for over a median follow‐up period of 102.1 months, was observed in 19 (41.3%) out of 46 patients. The 5‐year and 10‐year RFD‐free survivals were 61.2 and 47.2%, respectively. A hydronephrosis‐free condition was achieved in 76 (84.4%) out of 90 RUs. Stent insertion was identified as a significant predictive factor for post‐surgical RFD in CU, which indicated that the renal injury persisted even after the management of stomal obstruction of CU by stent insertion.

## AUTHOR CONTRIBUTIONS

**Chul Jang Kim**: Conceptualization; methodology; data curation; formal analysis; investigation; resources; writing—original draft; writing—review and editing. **Masayuki Nagasawa**: Statistical analysis; supervision. **Eiki Hanada**: Data curation; investigation. **Kayo Takeuchi**: Data curation. **Toshiyuki Ihara**: Data curation. **Susumu Kageyama**: Supervision; writing—review and editing.

## CONFLICT OF INTEREST STATEMENT

The authors declare that there are no conflicts of interest associated with this study.

## APPROVAL OF THE RESEARCH PROTOCOL BY AN INSTITUTIONAL REVIEWER BOARD

The present study was approved by the Institutional Review Board of Kohka Public Hospital (#445).

## INFORMED CONSENT

Informed consent was obtained through an opt‐out process.

## REGISTRY AND THE REGISTRATION NO. OF THE STUDY/TRIAL

Not applicable.

## ANIMAL STUDIES

Not applicable.
